# Assessment of effects of total sleep deprivation and subsequent recovery sleep: a methodological strategy feasible without sleep laboratory

**DOI:** 10.1186/s40359-021-00641-3

**Published:** 2021-09-15

**Authors:** Cindy Stroemel-Scheder, Stefan Lautenbacher

**Affiliations:** Department of Physiological Psychology, University of Bamberg, Markusplatz 3, Bamberg, Germany

**Keywords:** Total sleep deprivation, Recovery sleep, Portable polysomnography, Alertness, Fatigue

## Abstract

**Background:**

Sleep is critical for maintaining homeostasis in bodily and neurobehavioral functions. This homeostasis can be disturbed by sleep interruption and restored to normal by subsequent recovery sleep. Most research regarding recovery sleep (RS) effects has been conducted in specialized sleep laboratories, whereas small, less-well equipped research units may lack the possibilities to run studies in this area. Hence, the aims of the present study were to develop and validate an experimental protocol, which allows a thorough assessment of at-home recovery sleep after sleep deprivation.

**Methods:**

The experimental protocol, comprising one night of baseline sleep (BL) at home, one night of monitored total sleep deprivation and a subsequent recovery night at home, was tested in a sample of 30 healthy participants. Subjects’ fatigue and alertness were assessed prior to and after each night. Sleep at home (BL, RS) was objectively assessed using portable polysomnography. To check whether our at-home sleep assessments yielded results that are comparable to those conducted in sleep laboratories, we compared the sleep data assessed in our study with sleep data assessed in laboratory studies.

**Results:**

Sleep parameters assessed during RS exhibited changes as expected (prolonged total sleep time, better sleep efficiency, slow wave sleep rebound). Sleep parameters of BL and RS were in line with parameters assessed in previous studies examining sleep in a laboratory setting. Fatigue normalized after one night of RS; alertness partly recovered.

**Conclusions:**

Our results suggest a successful implementation of our new experimental protocol, emphasizing it as a useful tool for future studies on RS outside of well-equipped sleep laboratories.

## Background

Sleep is critical for maintaining homeostasis in bodily and neurobehavioral functions. Accordingly, recovery sleep (RS) after preceding sleep interruption is assumed to restore normal functioning of a wide range of measures, i.e., attention and sleepiness [1, 2], metabolism [3], immune function [4] and pain [5].

Most studies examining effects of recovery sleep have been conducted in specialized sleep centers and hospitals, which undoubtedly offers numerous advantages (e.g., standardized sleeping environments across participants, a thorough assessment of sleep via polysomnography, strict control of sleep deprivation and recovery). However, sleep laboratories provide a novel, unfamiliar sleeping environment including the presence of laboratory staff, which might lead to differences between laboratory sleep and habitual sleep at home [6–8]. Since sleep at home is assumed to especially promote restorative sleep [9], RS assessments at home might offer valuable and ecologically valid insights into sleep restoration. Up to now, there is nevertheless no experimental protocol available that—while meeting sufficiently high methodological standards—allows to assess RS in an at-home setting. Therefore, the aims of the present study were to develop and validate an experimental protocol that allows a thorough assessment of at-home recovery sleep.

For that purpose, we developed and tested an experimental protocol consisting of a night of habitual sleep (baseline, BL), a non-consecutive night of total sleep deprivation (TSD) and a night of subsequent recovery sleep (RS). TSD was guided and monitored in a regular office space at university. Sleep during BL and RS was objectively assessed using portable polysomnography, allowing subjects to sleep at home in a familiar sleeping environment. Daytime effects of habitual (BL) and recovery sleep (RS) as well as of sleep deprivation (TSD) on attention and fatigue were assessed using a cognitive test and a questionnaire before and after each night.

We hypothesized that sleep parameters of at-home sleep would show regular patterns during BL and compensatory changes during RS, i.e., prolonged total sleep time, slow wave sleep rebound, enhanced sleep efficiency as well as fewer and shorter awakenings during the night [10–12]. To further test the validity of our protocol, we additionally compared parameters of at-home sleep assessed in our study with sleep parameters assessed in laboratory studies. It appeared critical whether sleep parameters assessed at home were in a similar range as laboratory sleep parameters since strong deviations would have casted doubt on the validity of one or both methods of sleep assessments. Lastly, we expected fatigue and alertness to show a restoration to normal functioning following recovery sleep after a deterioration due to sleep deprivation [2, 10, 13], which would as well illustrate the validity of our protocol.

## Methods

### Participant enrollment and criteria for study participation

The present study was conducted at the University of Bamberg. Participants were recruited by advertisements at the University. Exclusion criteria were physical or mental disorders, acute or chronic pain, sleep disorders, shiftwork, surgery during the last six months, heavy smoking, current psychotherapy as well as current and regular medication intake (exception: oral contraceptives). Subjects were required to have a steady sleep–wake rhythm with habitual sleep durations of 7–9 h per night and regular times of going to bed and getting up in the morning. Further, these times of going to bed and getting up in the morning were required to not be shifted by more than 2 h during the weekend. Participating women were required to not be pregnant or nursing mothers. Ahead of the experiment, a short telephone-based interview was conducted with each participant to assess all above-mentioned inclusion and exclusion criteria; additionally, these criteria were re-assessed at the beginning of the first laboratory session.

**Fig. 1:**
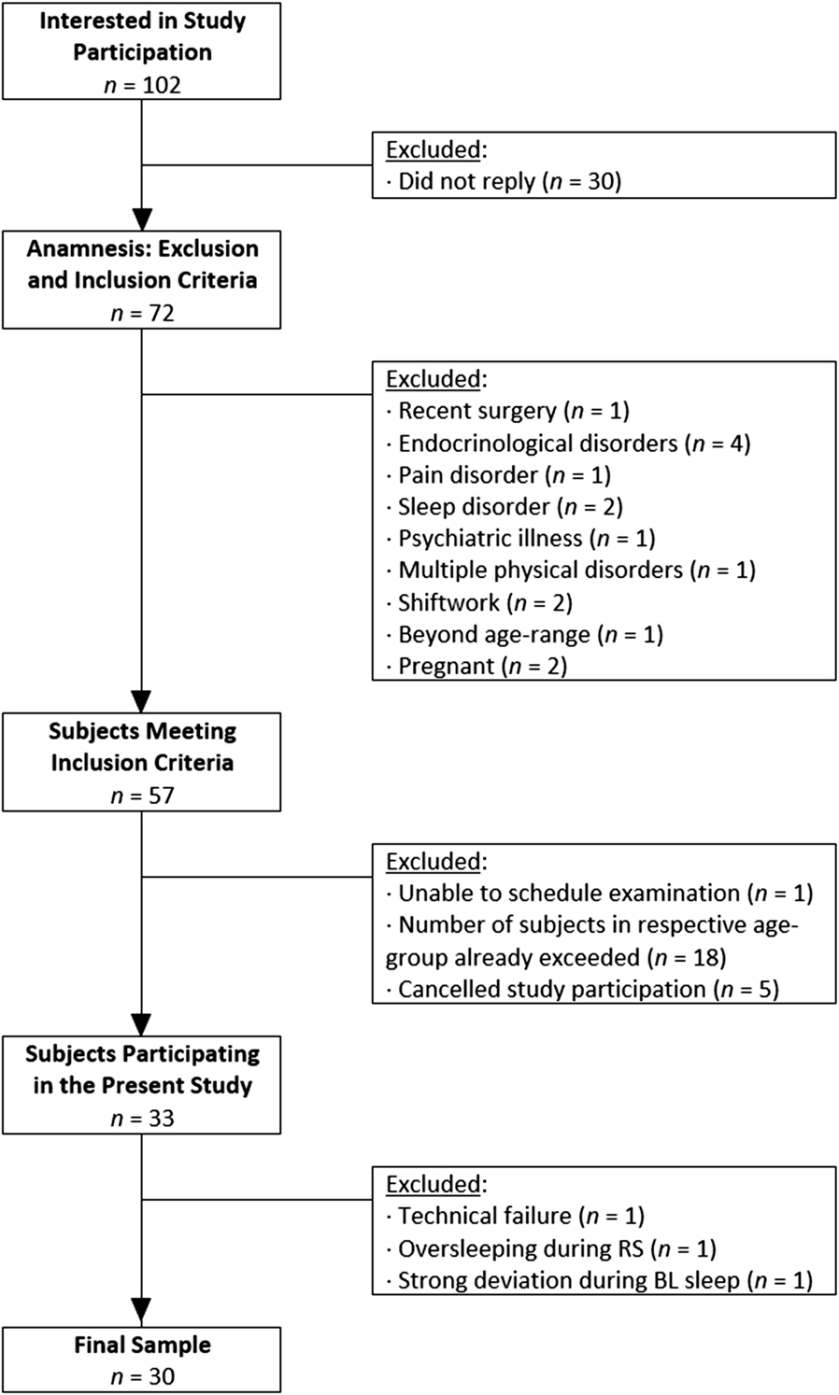
Flow-chart of participant enrollment

A detailed flow-chart depicting the participant enrollment of the current study can be found in Fig. 1; detailed sample characteristics will be reported in the results section. Overall, 30 participants (15 female) were included in the present study. All participants gave written informed consent and received monetary compensation or course credits (psychology students). The study was conducted in accordance with the Declaration of Helsinki; the experimental protocol gained ethical approval (Bayerische Landesärztekammer, Munich, Germany; #17037).

### General protocol

**Fig. 2:**
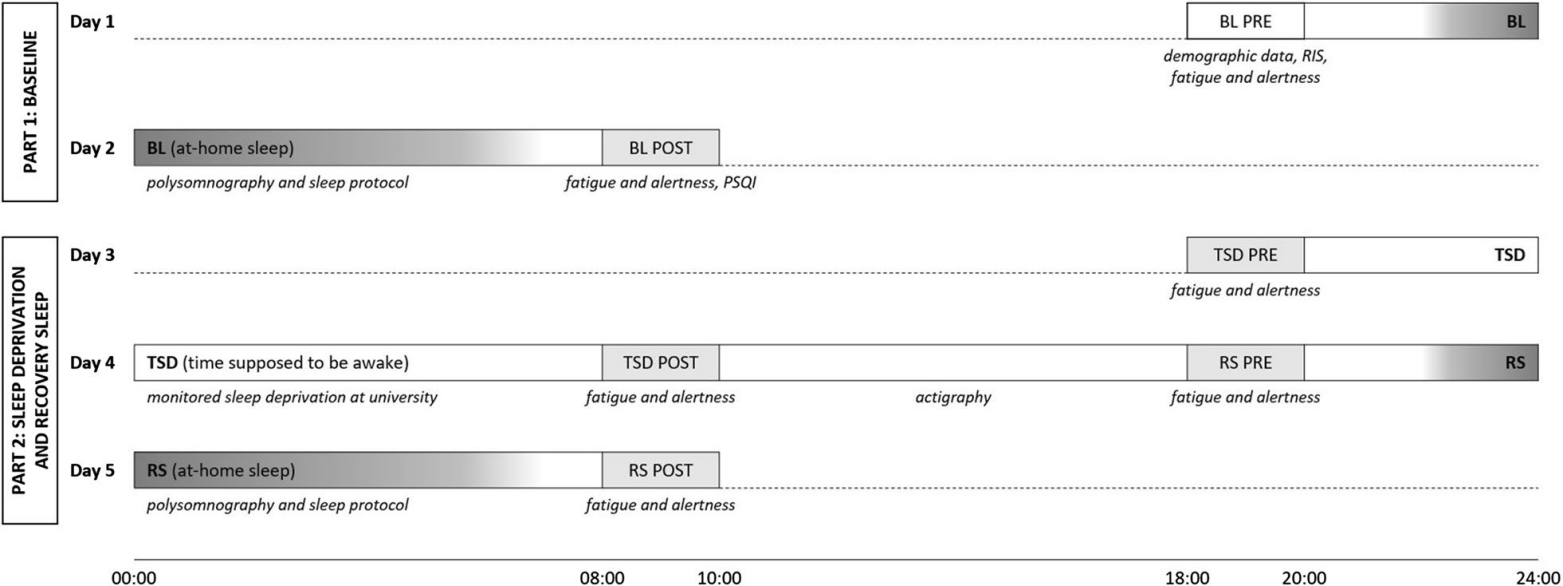
Schematic overview of the study design. The study comprised two parts, namely a baseline part (habitual sleep, BL) and a part with experimental sleep manipulations (total sleep deprivation, TSD; recovery sleep, RS). Laboratory sessions, as indicated by boxes in light gray, were conducted the evening before each night (18:00, denoted as “PRE”) and the morning after each night (08:00, denoted as “POST”). During all six laboratory sessions, fatigue was assessed using the POMS (Profile of Mood States) and alertness was assessed using the TAP (Test Battery for Attentional Performance). Sleep, as indicated by bars in dark gray, was assessed using portable polysomnography (PSG) and a self-report questionnaire (evening- and morning-protocols). RIS = Regensburg Insomnia Scale. PSQI = Pittsburgh Sleep Quality Index

In this paragraph, an overview of the general protocol of the study will be provided in chronological order (refer to Fig. 2 for a schematic overview). In the paragraphs following thereafter, all measures will be described in detail (see “Manipulation check” and “Dependent variables” sections).

In short, the study comprised two parts: part one included a baseline night (BL) of habitual sleep at home (see “Baseline assessments” section); part two included one night of total sleep deprivation (TSD) and a night of subsequent recovery sleep (RS) at home (see “Total sleep deprivation and recovery sleep” section). In the following, the three assessed nights are referred to as “BL” (baseline night), “TSD” (night of total sleep deprivation) and “RS” (night of recovery sleep). Laboratory testing sessions, during which fatigue and alertness were assessed, were conducted on the evening prior to and in the morning after each night. Evening laboratory sessions were conducted at 18:00 (PRE) and morning laboratory sessions at 08:00 (POST). During the whole study period, participants were required to refrain from consuming alcohol and taking medication as well as to not change their regular habits regarding the intake of caffeinated beverages, smoking and physical activity. Participants had to strictly avoid daytime naps. Further, participants were not investigated in the week following change to daylight saving time or return to standard time.

#### Baseline assessments

##### BL PRE

Subjects arrived at a laboratory of psycho-physiological testing at 18:00 in the evening for their first laboratory assessment (BL PRE). All relevant information for study participation—which was already given prior to the subjects’ first laboratory session in written form—was now repeated in detail. Afterwards, participants provided written informed consent. In a next step, the following demographic and clinical variables were assessed: age, sex, height, weight, use of optical aids, tobacco use, highest educational achievement as well as the above listed inclusion and exclusion criteria (see “Participant enrollment and criteria for study participation” section). Participants then completed the Regensburg Insomnia Scale (RIS) [14], a short self-report rating scale of ten items to assess psychological symptoms and sleep in insomnia. It asks for the time subjects usually go to bed and get up, the time they need to fall asleep (sleep latency), how many hours they sleep during the night, sleeping problems (e.g., disturbed sleep, waking up too early) and whether they feel fit during the day. The RIS-score can range between a minimum of 0 and a maximum of 40 points (cut-off: 12 points), with higher scores indicating worse sleep quality.

Afterwards, the assessment of subjects’ fatigue and alertness followed (see “Dependent variables” section for a detailed description). In short, participants completed the Profile of Mood States (POMS), which allowed an assessment of their momentary level of fatigue. They then completed the subtest “alertness” of the Test Battery of Attentional Performance, which allowed an assessment of tonic and phasic alertness.

After completing these tests, participants were equipped with a portable polysomnography (PSG) device (see “Portable polysomnography (PSG)” section) to assess objective parameters of at-home sleep. First, subjects were asked to put their pajama shirt on; then, electrodes were attached to the participants’ head and face. Next, a shoulder and chest strap were attached to the subjects’ upper body to hold the PSG-device in place. After checking that the electrodes and straps were well fastened, subjects were instructed to avoid strain on the cables. They were handed out evening- and morning-protocols to assess subjective sleep parameters and sleep quality (see “Evening- and morning-protocols” section). Participants then left the laboratory.

##### BL

Subjects spent their baseline night of habitual sleep at home in their familiar sleeping environment. The BL-night helped to ensure that participants exhibited regular sleep patterns. Sleeping times of subjects were solely restricted by the requirement to come to the laboratory on time in the morning.

##### BL POST

During the morning session (BL POST at 08:00), the experimenter first carefully detached the PSG-device and collected the evening- and morning-protocols. Afterwards, a next assessment of fatigue and alertness followed. Thereafter, the subjects completed the Pittsburgh Sleep Quality Index (PSQI) to report habitual sleep characteristics during the past month [15]. The PSQI consists of 19 self-report items, which are answered on a four-point rating scale (ranging from 0 to 3 points). The PSQI asks for the time subjects usually go to bed and get up, sleep onset latency and sleep duration, problems with sleep (e.g., delayed sleep onset, frequent awakenings) and sleep quality. Overall, seven component scores (sleep quality, sleep latency, sleep duration, sleep efficiency, sleep disorders, sleep medication use, daytime sleepiness) and one global score can be derived. The global score can range between a minimum of 0 and a maximum of 21 points, with higher scores indicating worse sleep quality.

#### Total sleep deprivation and recovery sleep

The night of habitual sleep (BL) and the night of total sleep deprivation (TSD) were not consecutively conducted; in the present study, both nights (BL and TSD) were between one and four nights apart.

##### TSD PRE

Subjects arrived at the laboratory at 18:00 in the evening for their evening assessment, during which fatigue and alertness were again assessed. After finishing the laboratory session (circa 19:00) subjects were taken to an office at the university where the total sleep deprivation procedure was conducted. In the office room, the TSD-procedure was explained to the participants, and they were given the opportunity to familiarize themselves with the office room. The room was equipped with desks, chairs, a sofa, a computer, a TV, a game console, a fridge, dishes, cutlery as well as a water kettle, water, and tea.

##### TSD

During the night of total sleep deprivation (20:00–08:00), sleep was fully prevented. To ensure wakefulness of the participants, they were closely monitored one-on-one by experimenters during the entire night. To ensure vigilance on the side of the monitoring staff, each TSD-night was run in two shifts by two experimenters (first shift from 20:00 to 02:00, second shift from 02:00 to 08:00; shifts alternated between the two experimenters to balance the strain by night work). The night of TSD followed a standardized procedure consisting of activities with varying activity-levels (see Table 1). This procedure was chosen based on previous sleep deprivation studies [16] and was adapted to be viable outside a clinical setting. In case of a subject reporting excessive tiredness or beginning to doze off, the experimenter chose an activity with a higher activity-level. Experimenters recorded the activities conducted during the night of TSD, also indicating the respective activity-levels and events of relevance (e.g., when an activity with higher activity-level was performed in deviation from the protocol because a participant reported excessive tiredness). At 07:00 participants received a standard-breakfast (bun with honey, cup of fruit tea or herbal tea). If necessary, subjects were allowed to take a short walk prior to their morning laboratory session TSD POST, which started at 08:00.

**Table 1:** Protocol of the activities conducted during total sleep deprivation (TSD) and the respective activity-levels

Time	Activity	Physical activity-level
20:00–22:00	Dinner	Low
	Watching movie	Low
22:00–00:00	Watching/finishing movie	Low
	Parlor games	Low
00:00–02:00	One-hour walk	Moderate to high
	Console games	Moderate to high
02:00–04:00	Console games	Moderate to high
	Parlor games	Low
04:00–06:00	One-hour walk	Moderate to high
	Console games	Moderate to high
	Talking, watching movie	Low
06:00–07:55	Watching/finishing movie	Low
	Standard breakfast	Low
	(If necessary: short walk)	Moderate

The total sleep deprivation procedure started after the TSD PRE laboratory session. At 08:00 the morning laboratory session (TSD POST) followed

##### TSD POST

During the morning laboratory session (TSD POST at 08:00), fatigue and alertness were assessed. At the end of the laboratory session, subjects were instructed to spend their day as usual, but to avoid naps, to avoid driving motor vehicles and not to do any activities requiring high levels of attention. They were equipped with an actigraph and were handed out a booklet that included guidelines for their behavior, guidelines for how to handle the actigraph and questions regarding the subjects’ day (see “Actigraphy” section for further details). Subjects then left the laboratory.

##### RS PRE

Subjects arrived at the laboratory at 18:00 in the evening for their evening assessment (RS PRE). This laboratory session was the session during which the subjects were awake for the longest continuous time. First, the actigraph was detached and the booklet was collected by the experimenter. The assessment of fatigue and alertness followed. Then, participants were equipped with the portable PSG-device and were handed out the evening- and morning-protocols (same procedure as described for BL PRE). Participants then left the laboratory to sleep at home.

##### RS

Subjects were allowed to spend their recovery night (RS) at home in a familiar sleeping environment. As during their baseline-night (BL), subjects’ sleeping times were again solely restricted by the requirement to come to the laboratory on time in the morning (08:00).

##### RS POST

During the morning laboratory session (RS POST at 08:00), the experimenter detached the PSG-device and collected the evening- and morning-protocols. Afterwards, a final assessment of fatigue and alertness followed, upon which study participation ended.

### Manipulation check

#### Portable polysomnography (PSG)

##### Apparatus and protocol

For PSG-recordings, the SOMNOwatch™ plus EEG6 (SOMNOmedics, Randersacker, Germany) [17] was used, which proved to be a feasible recorder for at-home sleep measurements in previous studies [18, 19]. PSG-recordings were prepared in the laboratory at the end of BL PRE and RS PRE (evening laboratory sessions prior to the subjects’ baseline night and recovery night, respectively). Prior to attaching the PSG-device subjects were allowed to put their pajama-shirt on since a change of clothes was not possible after the PSG-device was attached. Four EEG-channels (C3, C4, O1, O2), two channels of electrooculogram (EOG; first channel above left eye and second channel underneath right eye diagonally to first channel) and two channels of electromyogram (EMG, M. submentalis) were recorded using gold disc electrodes (Grass Technologies, West Warwick, USA). A ground electrode was fixed at the forehead. EEG-electrodes were positioned based on the international 10–20-system. All nine electrodes were referenced towards Cz. To ensure a best possible attachment of electrodes and to reduce electrode resistance, the skin was carefully cleaned and peeled with cleansing gel (Nuprep, Weaver and Company, Aurora, CO, USA). Further, an electrode cream was applied (EC2 Electrode Cream, Grass Technologies, West Warwick, USA). To ensure a thorough attachment of EEG-electrodes to the skin, the participants’ hair was carefully parted. Finally, all electrodes were fixed at the participants’ head using a piece of mull and a fixing plaster (Fixomull Stretch, BSN Medical GmbH, Hamburg, Germany).

Thereafter, the fastening of all electrodes was checked. Further, it was checked that the shoulder and chest strap, which were used to hold the PSG-device in place at the subjects’ chest, were well fastened but did not press. To avoid strain on the cables and to avoid that the subjects’ hair or clothes tangle with the cables, cables were loosely tied together using a fixing plaster. Subjects were asked to avoid pulling on the cables, to not wear any headgear and to not pull clothes with tight collars over their head. Then, subjects left the laboratory and slept at home (during BL and RS). They were instructed to follow their regular sleeping habits.

##### Evaluation

PSG-recordings were analyzed according to the standard PSG-protocol [20]. The DOMINO light software (SOMNOmedics, Randersacker, Germany) automatically scored sleep and wake stages in epochs of 30 s. Afterwards, a visual inspection was performed by a trained examiner to check whether the automatic analysis correctly scored stages according to the Rechtschaffen and Kales criteria. In case of discrepancy, the automatic analysis was overruled, and stages were rescored.

##### Measures

Parameters derived from PSG-recordings and used for further examination were divided into general and sleep-stage specific parameters. As general PSG parameters, total sleep time (TST, time between “lights off” and “lights on” without sleep onset latency and time spent awake), sleep efficiency (SE, total sleep time / time spent in bed after sleep onset * 100%), sleep onset latency (SL, time from “lights off” to the first appearance of non-REM 2) and the total number and duration of awakenings were used. Sleep-stage specific PSG parameters were absolute and relative (percentage of TST) durations of non-REM 1, non-REM 2, slow wave sleep (SWS; non-REM stages 3 and 4) and rapid-eye-movement sleep (REM sleep).

#### Evening- and morning-protocols

Evening- and morning-protocols of the German Sleep Society are sleep logs frequently used to assess self-reported parameters of sleep in German speaking samples [21]. These sleep logs show a satisfactory validity and reliability [21]. They were used to assess self-reported sleep characteristics and sleep quality during BL and RS. In the present study, the protocols were handed out at the end of the evening laboratory sessions prior to the subjects’ baseline night and recovery night (BL PRE and RS PRE, respectively); the next mornings, the protocols were collected by the experimenter (during BL POST and RS POST, respectively).

The evening-protocol, which is filled in directly before going to bed, consists of eight questions asking for the subjects’ current mood, freshness, and tension, how productive they had felt during the day, how exhausted and fatigued they had felt during the day, whether they had slept during the day (if so: when and how long) and if there were any strains during the day. Also, their intake of drinks and tobacco was assessed, as well as the time they went to bed. Lastly, an open question is included to give subjects the opportunity to indicate in own words, if something unusual had happened during their day. The morning-protocol, which is completed immediately after waking up in the morning, includes ten questions. It asks for subjects’ current mood, freshness and tension, restfulness of sleep, how long subjects were in bed before turning the lights off, how long it took them to fall asleep, and how often and how long they were awake during the night. Subjects could as well indicate whether they slept badly and for what reason. Further it is asked for whether subjects dreamt at night, the time they woke up and how (with/without an alarm clock), how long they slept, when they got up in the morning and whether they took medication.

##### Measures

For a further evaluation of self-reported sleep quality during BL and RS, four items of the morning-protocol were used. Three items dealing with mood (ranging from “depressed” to “untroubled”), freshness (ranging from “run down” to “refreshed”) and tension (ranging from “tense” to “relaxed”) were answered on a 6-point scale. A question about restfulness of sleep was answered on a 5-point scale (ranging from “very restful” to “not restful at all”). These items served as parameters of subjective sleep quality.

#### Actigraphy

For actigraphic recordings, the SOMNOwatch™ (SOMNOmedics, Randersacker, Germany) was again used, which is a device that can be used for both polysomnographic and actigraphic recordings. The device recorded and stored the acceleration in three axes (x-axis, y-axis, z-axis) as a magnitude signal. It was attached to the subjects’ wrist (non-dominant arm) with a soft strap with Velcro.

After attaching the actigraph, participants received a detailed instruction of how they were allowed to spend their day after their night of total sleep deprivation (to spend their day as usual, but to not drive motor vehicles, to not do any activities requiring high levels of attention and to strictly avoid naps). They were further instructed to detach the actigraph when they showered or bathed; otherwise, the device had to be worn the whole day. In this regard, subjects were handed out a booklet that included (1) the guidelines for their behavior, (2) guidelines for how to handle the actigraph (detach the actigraph while showering/bathing, not exposing the device to high heat, cold or humidity) and (3) questions regarding the subjects’ day. These questions were “did you detach the actigraph?” (yes/no) and, if yes, at what time, for how long, and for what reason. A further question was “did you sleep during the day?” (yes/no) and, if yes, at what time and for how long. Lastly, an open question “did something of relevance happen today that you would like to tell the experimenters?” was included to allow participants to report notable events that had happened during the day. On- and offset of the activity of subjects (as measured by the magnitude signal of the actigraph) and the times in which the actigraph was reportedly detached were compared with one another to check for agreement.

### Dependent variables

Dependent variables were assessed during laboratory sessions conducted in a laboratory for psycho-physiological testing at the University of Bamberg prior to (18:00; PRE) and after (08:00; POST) each night, resulting in overall six laboratory sessions per participant.

#### Fatigue

A short version of the POMS (Profile of Mood States) consisting of 35 adjectives (e.g., active, tense, lively) was assessed [22–24]. According to their current mood, participants rated these adjectives on a 7-point scale ranging from “0—not at all” to “6—extremely strong”. The questionnaire provides four distinct mood subscales (depression/anxiety (14 items), vigor (7 items), fatigue (7 items), hostility (7 items)) and a total score (total mood disturbance). In a psychometric evaluation in a representative German sample, the POMS yielded satisfactory internal consistency [23]; the same result was found in studies assessing the English short form of the POMS in English-speaking samples [25, 26].

##### Measures

Only the subscale “fatigue”, which consists of seven adjectives (worn-out, listless, fatigued, exhausted, sluggish, weary, bushed) was used for further evaluation as a measure of subjects’ current fatigue. The POMS fatigue-score can range between a minimum of 0 and a maximum of 42 points, with higher scores indicating stronger current fatigue.

#### Alertness

The Test Battery of Attentional Performance (TAP) [27] is a standardized and computerized neuro-psychological test. To assess attention, the subtest “alertness” of the TAP version 2.3 was used. In its implementation and evaluation, the TAP is objective; further, the subtest “alertness” yielded a high reliability [27].

The subtest “alertness” consists of two conditions which are repeated twice. In a first condition, simple reaction times are assessed as a measure of “tonic alertness” (task without warning stimulus prior to a visual target stimulus (a cross on a computer screen in front of subjects)). In a second condition, cued reaction times are assessed as a measure of “phasic alertness” (task with warning stimulus (acoustic warning signal) prior to the same visual target stimulus). Tonic alertness reflects the general speed of information processing by assessing reaction times to simple attention tasks and phasic alertness reflects the degree by which a warning signal (prior to the target stimulus) can increase this speed of reacting [28]. Both tasks refer to the intensity of attention and are sensitive to the level of vigilance.

During the task, participants sat with their back to the experimenter to minimize stress due to being watched; nevertheless, the experimenter could monitor subjects by using a webcam in front of them. This allowed the experimenter to check that participants remained continuously awake during the task (which was especially important after sleep deprivation during TSD POST and RS PRE) and to immediately intervene if they fell asleep.

##### Measures

The TAP subtest “alertness” yields information about means, medians as well as standard deviations of both cued and un-cued reaction times. For further evaluation, median reaction times without warning signals as a measure of tonic alertness, their standard deviations as a measure of attentional variability, and median reaction times with warning signals as a measure of phasic alertness were used.

### Statistical analyses

Data were analyzed using SPSS version 25 (SPSS Inc., Chicago, IL, USA). Significance level was set at α = 5%. Data are presented as mean and standard deviation. Bonferroni corrections for multiple testing were applied. To check for differences in sleep nights (BL vs. RS), separate multivariate analyses of variance (MANOVAs) were conducted with within-subject factor “night” (BL, RS) for “general PSG” (TST, SE, SL, total number and duration of awakenings), “sleep-stage specific PSG” (non-REM 1, non-REM 2, SWS and REM sleep; considering both absolute durations and percentages of sleep stages relative to total sleep time in separate MANOVAs), and “subjective sleep quality” (mood, freshness, tension, restfulness). To check for differences in sleep effects on alertness and fatigue, repeated measurement ANOVAs were conducted with the within-subject factors “condition” (BL, TSD, RS) and “time of day” (evening (PRE), morning (POST)). Post-hoc t-tests were computed for detailed analyses. For F-tests partial eta-squared (*η*^2^) is reported as an estimate of effect size. Cohen’s *d* is reported to describe effect sizes for paired comparisons.

## Results

In the following paragraphs, an illustration of sample characteristics as well as results concerning the effects of the experimental manipulations on parameters of sleep assessed during BL and RS will be reported. Then, to illustrate the validity of our protocol, a comparison of at-home sleep parameters (as assessed in our study) with sleep parameters of laboratory studies with a similar design follows. Lastly, effects of recovery sleep on fatigue and alertness will be reported and aspects of subjects’ compliance as well as technical considerations will be illustrated.

### Sample characteristics

In the present study, a sample of 33 healthy subjects was assessed, of which three participants had to be excluded (see Fig. 1). One subject reported falling asleep for approximately 5 min, which was seen in actigraphy; nevertheless, due to the short nap duration and since the results of this subject did not strongly deviate from the rest of the group, this subject was nonetheless included in all analyses. Thus, 30 subjects (15 female) with an age of *M* = 33.70 years (*SD* = 10.47; *range* = 19–55 years) were included. Demographic data and scores of sleep questionnaires (RIS, PSQI)—which indicate that the subjects examined in the present study were good sleepers—can be found in Table 2.

**Table 2:** Demographic data and questionnaire-scores

*Demographic data*	
Sample size	*n* = 30
Male/female	*n* = 15/*n* = 15
Age (years)^a^	33.70 (10.47)
Age range (years)	19–55
*Sleep duration*	
Habitual sleep duration (hours)^a,b^	7.40 (0.95)
*Sleeping environment*	
Bedpartners^c^	
Without Bedpartner	*n* = 15
With Bedpartner	*n* = 15
*Questionnaire scores*	
RIS^a^	6.50 (3.33)
PSQI^a^	
Sleep quality	0.80 (0.41)
Sleep latency	0.83 (0.87)
Sleep duration	0.20 (0.41)
Sleep efficiency	0.33 (0.61)
Sleep disorders	1.07 (0.25)
Sleep medication use	0.07 (0.25)
Daytime sleepiness	0.83 (0.38)
Overall PSQI-score	4.13 (1.57)

RIS = Regensburg Insomnia Scale, PSQI = Pittsburgh Sleep Quality Index. RIS-scores can vary between 0 and 40 points. Scores of PSQI-subscales can vary between 0 and 3 points, the overall PSQI-score can vary between 0 and 21 points

^a^Results are presented as *M* (*SD*)

^b^Data derived from PSQI-item “During the past month, how many hours of actual sleep did you get at night?”

^c^Data derived from PSQI-item “Do you have a bedpartner or roommate?”

### Effects of experimental manipulations on sleep

As expected, a significant effect of factor “night” was found for “general PSG” (*F*_5,54_ = 25.554, *p* < 0.001, *η*^2^ = 0.703) in the MANOVA comparing BL and RS. Recovery sleep was characterized by a significantly longer sleep time, better sleep efficiency, a faster sleep onset as well as shorter and fewer awakenings (see Table 3). Also, a significant effect of factor “night” was found for “sleep-stage specific PSG” considering absolute values (*F*_4,55_ = 27.756, *p* < 0.001, *η*^2^ = 0.669) and durations relative to TST (*F*_3,56_ = 5.037, *p* = 0.004, *η*^2^ = 0.213) in the MANOVAs comparing BL and RS. In line with the prolonged total sleep time, the absolute amount of time spent in non-REM 2, REM and SWS was significantly prolonged during RS (see Table 3). Whereas the percentage of SWS was significantly increased during RS, the percentage of non-REM 1 was decreased (see Table 3). Lastly, a significant effect of factor “night” was found for subjective sleep quality (*F*_4,55_ = 3.679, *p* = 0.010, *η*^2^ = 0.211) in the MANOVA comparing BL and RS. There were no differences between BL and RS according to mood, freshness, and tension; however, self-reported restfulness of sleep was significantly higher after RS (*t* = − 3.525, *p* < 0.001, *d* = 0.794) (see Table 3).

**Table 3:** Descriptive data of sleep parameters and results of post-hoc comparisons

	Descriptive data		Post-hoc comparisons		
	BL	RS	t	*p*	d
*General PSG*					
Total sleep time^a^	**6:59:00 (0:52:59)**	**9:41:21 (1:08:18)**	− **13.972**	**< 0.001**	**2.657**
Sleep efficiency %	**94.20 (6.39)**	**98.18 (2.74)**	− **4.840**	**< 0.001**	**0.811**
Sleep onset latency^a^	**0:14:08 (0:11:38)**	**0:03:52 (0:03:45)**	**4.852**	**< 0.001**	− **1.193**
Time awake^a^	**0:38:52 (0:30:53)**	**0:13:50 (0:16:36)**	**6.574**	**< 0.001**	− **1.008**
Nb. of awakenings	**4.00 (2.18)**	**2.37 (1.45)**	**3.797**	**0.001**	− **0.880**
*Sleep-stage specific PSG (absolute durations)*					
Duration non-REM 1^a^	0:28:15 (0:08:23)	0:29:42 (0:12:22)	− 0.800	0.430	0.137
Duration non-REM 2^a^	**3:55:42 (0:44:46)**	**5:17:47 (0:58:57)**	− **9.609**	**< 0.001**	**1.568**
Duration REM^a^	**1:08:56 (0:21:15)**	**1:27:16 (0:30:58)**	− **4.010**	**< 0.001**	**0.689**
Duration SWS^a^	**1:26:07 (0:24:53)**	**2:26:35 (0:44:00)**	− **9.964**	**< 0.001**	**1.532**
*Sleep-stage specific PSG (percentage relative to TST)*					
Percent non-REM 1^b^	**6.85 (2.28)**	**5.15 (2.14)**	**5.010**	**< 0.001**	− **0.769**
Percent non-REM 2^b^	56.03 (5.91)	54.72 (7.76)	1.346	0.189	− 0.189
Percent REM^b^	16.60 (5.06)	14.92 (4.73)	2.107	0.044	− 0.343
Percent SWS^b^	**20.53 (5.28)**	**25.22 (6.99)**	− **5.408**	**< 0.001**	**0.757**
*Subjective sleep quality*					
Mood^c^	4.47 (0.97)	4.33 (1.06)	0.660	0.514	− 0.138
Freshness^d^	3.53 (1.07)	3.67 (1.32)	− 0.571	0.573	0.117
Tension^e^	4.40 (1.04)	4.33 (1.03)	0.284	0.778	− 0.068
Restfulness of sleep^f^	**3.40 (0.62)**	**4.00 (0.87)**	− **3.525**	**0.001**	**0.794**

Results are presented as *M* (*SD*). *df* = 29. Bonferroni corrected α’s: General PSG α = 0.01, Sleep-Stage Specific PSG α = 0.0125, Subjective Sleep Quality α = 0.0125. Significant differences are marked in bold

^a^hours:minutes:seconds

^b^Percental amounts of sleep stages as relative to total sleep time (TST)

^c^Mood, ranging from “1—depressed” to “6—untroubled”

^d^Freshness, ranging from “1—run down” to “6—refreshed”

^e^Tension, ranging from “1—tense” to “6—relaxed”

^f^Restfulness, ranging from “1—not restful at all” to “5—very restful”

To summarize the main findings regarding sleep, RS was characterized by a significantly longer sleep time, better sleep efficiency, a faster sleep onset as well as shorter and fewer awakenings. Notably, a significant increase in slow wave sleep was found for both absolute as well as relative SWS durations during RS as compared to BL, highlighting a profound SWS rebound. Self-reported sleep quality was similar during BL and RS; solely self-reported restfulness of sleep was higher during RS.

### Comparison of at-home sleep and laboratory sleep

In the following, a comparison of sleep parameters assessed in our study of at-home sleep with sleep parameters assessed in laboratory studies follows. A detailed overview of parameters of baseline sleep and recovery sleep of our study in comparison to parameters assessed in four laboratory studies [10, 11, 29, 30] is provided in Tables 4 and 5. The laboratory studies were chosen based on their methodological similarity to our study since they included one or several baseline nights, a night of sleep deprivation and one or more nights of recovery sleep. To allow a comparison of at-home sleep and laboratory sleep, Tables 4 and 5 give detailed numerical values of assessed sleep parameters (descriptive data and effect sizes); the laboratory sleep data can thus be seen as a reference for the comparison of at-home and laboratory sleep.

**Table 4:** Comparison of general PSG parameters of at-home sleep and laboratory sleep

	At-home sleep			Laboratory sleep											
				Study 1			Study 2			Study 3			Study 4		
	BL	RS		BL	RS		BL	RS		BL	RS		BL	RS	
	M (SD)	M (SD)	d	M (SD)^a^	M (SD)^a^	d	M (SD)^a^	M (SD)^a^	d	M (SD)	M (SD)	d	M (SD)	M (SD)	d
*Total sleep time (minutes)*	419.00 (52.98)	581.35 (68.30)	2.657	–	–	–	411 (29.93)	543 (22.45)	4.989	449.75 (22.67)	445.06 (17.22)	− 0.233	449.61 (21.21)	447.56 (17.76)	− 0.105
*Sleep efficiency %*	94.20 (6.39)	98.18 (2.74)	0.811	–	–	–	85 (7.48)	94 (3.74)	1.522	92.76 (4.89)	97.18 (0.91)	1.257	92.54 (4.62)	97.24 (0.87)	1.414
*Sleep onset latency (minutes)*	14.13 (11.63)	3.87 (3.75)	− 1.193	12.0 (4.53)	4.0 (3.39)	− 2.000	25 (14.97)	6 (3.74)	− 1.741	16.31 (15.43)	3.68 (2.31)	− 1.145	19.11 (16.70)	4.06 (2.43)	− 1.261
*Time awake (minutes)*	38.87 (30.88)	13.83 (16.60)	− 1.008	6.8 (9.05)	1.0 (1.13)	− 0.899	47 (22.45)	27 (22.45)	− 0.891	18.31 (10.28)	9.75 (2.99)	− 1.131	17.33 (7.55)	9.33 (4.26)	− 1.305

The table gives information about at-home sleep parameters as assessed in our study (values are presented as M (SD); see also Table 3). Sleep as assessed in laboratory studies is reported for four studies (values are presented as M (SD)). Study 1: Achermann et al. [29]; Study 2: Arnal et al. [10]; Study 3: Curcio et al. [11]; Study 4: De Gennaro & Ferrara [30]. As effect size Cohen’s *d* is reported

^a^In these studies, descriptive data was given as *M* ± *SEM*, thus, *SD* was calculated by multiplying *SEM* by the square root of *N* (sample size)

**Table 5:** Comparison of sleep-stage-specific parameters of at-home sleep and laboratory sleep

	At-home sleep			Laboratory sleep											
				Study 1			Study 2			Study 3			Study 4		
	BL	RS		BL	RS		BL	RS		BL	RS		BL	RS	
	M (SD)	M (SD)	d	M (SD)^a^	M (SD)^a^	d	M (SD)^a^	M (SD)^a^	d	M (SD)	M (SD)	d	M (SD)	M (SD)	d
Duration non-REM 1 (minutes)	28.25 (8.38)	29.70 (12.37)	0.137	34.3 (12.73)	18.1 (7.64)	− 1.543	20 (7.48)	7 (3.74)	− 2.198	32.93 (11.13)	19.74 (8.95)	− 1.306	–	–	–
Duration non-REM 2 (minutes)	235.70 (44.77)	317.78 (58.95)	1.568	–	–	–	175 (33.68)	193 (33.68)	0.534	257.94 (26.83)	222.99 (38.28)	− 1.057	–	–	–
Duration SWS (minutes)	86.12 (24.88)	146.58 (44.00)	1.532	90.4 (19.80)	136.2 (24.33)	2.065	132^b^ (26.19)	231^b^ (48.64)	2.534	54.87 (20.70)	102.13 (44.18)	1.370	–	–	–
Duration REM (minutes)	68.93 (21.25)	87.27 (30.97)	0.689	–	–	–	84 (22.45)	112 (33.68)	0.978	103.99 (23.72)	100.19 (19.90)	− 0.174	–	–	–

The table gives information about at-home sleep parameters as assessed in our study (values are presented as M (SD); see also Table 3). Sleep as assessed in laboratory studies is reported for four studies (values are presented as M (SD)). Study 1: Achermann et al. [29]; Study 2: Arnal et al. [10]; Study 3: Curcio et al. [11]; Study 4: De Gennaro & Ferrara [30]. As effect size Cohen’s *d* is reported

REM = Rapid-Eye-Movement Sleep. SWS = Slow Wave Sleep (stages non-REM 3 and 4)

^a^In these studies, descriptive data was given as *M* ± *SEM*, thus, *SD* was calculated by multiplying *SEM* by the square root of *N* (sample size)

^b^In this study, values for the duration of non-REM 3 were reported

Regarding the different sleep parameters, there was a large variability across the laboratory studies. Considering general PSG parameters, the total sleep time during baseline was comparable between at-home and laboratory sleep. During the subjects’ recovery night at home, they were able to sleep markedly longer as compared to laboratory studies, what reflects a longer sleep opportunity at home as compared to laboratory settings.

There were no pronounced differences regarding sleep efficiency (SE), sleep latency (SL), the time subjects spent awake during the night between as well as the durations of specific sleep stages between at-home and laboratory sleep considering both baseline and recovery sleep. The most prominent finding was a similarity regarding a slow wave sleep rebound during both at-home and laboratory recovery sleep. In summary, there was considerable variability in sleep parameters across laboratory studies; a variability, in which the sleep parameters assessed in our study of at-home sleep fit in.

### Effects of experimental manipulations on fatigue and alertness

#### Fatigue

**Fig. 3:**
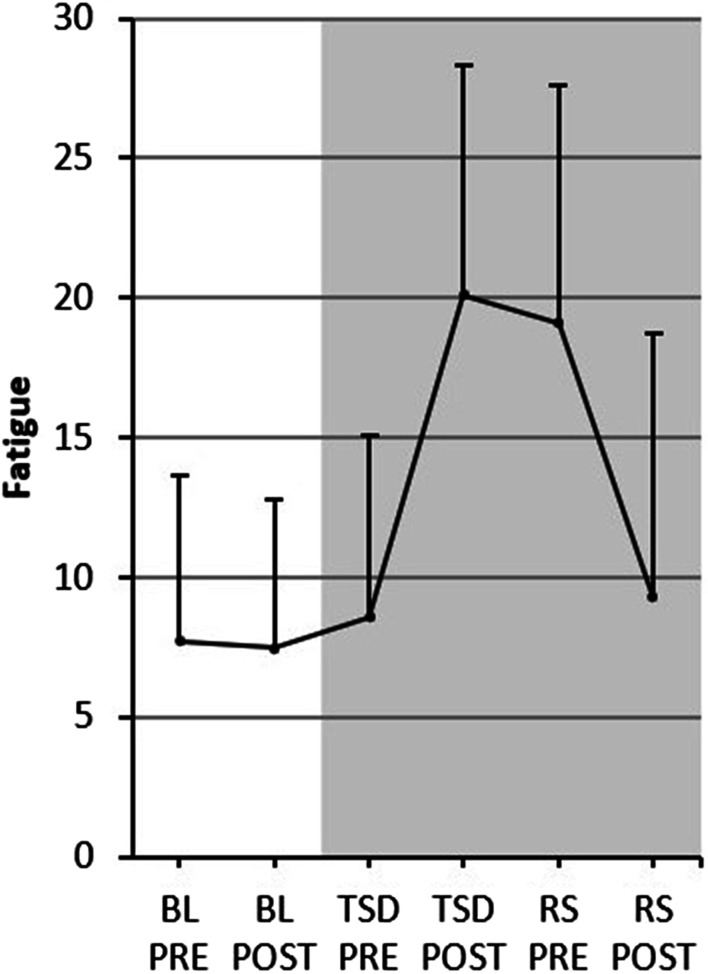
Fatigue-score as measured by the Profile of Mood States (POMS) subscale “fatigue”. Results are presented as *M* and *SD*. The fatigue-score can range between *min* = 0 and *max* = 42

Detailed fatigue-scores can be found in Fig. 3. The ANOVA yielded a significant effect of factor “condition” (*F*_2,28_ = 51.602, *p* < 0.001, *η*^2^ = 0.787) and a significant interaction “condition * time of day” (*F*_2,28_ = 38.817, *p* < 0.001, *η*^2^ = 0.735). Subjects reported significantly higher fatigue after sleep deprivation as compared to values assessed prior to sleep deprivation (TSD PRE vs RS PRE: *t*_29_ = − 6.533, *p* < 0.001, *d* = 1.387). Fatigue was significantly lower after recovery sleep as compared to post-deprivation values (TSD POST vs RS POST: *t*_29_ = 7.291, *p* < 0.001, *d* = − 1.215), which is indicative of a full recovery.

#### Alertness

**Fig. 4:**
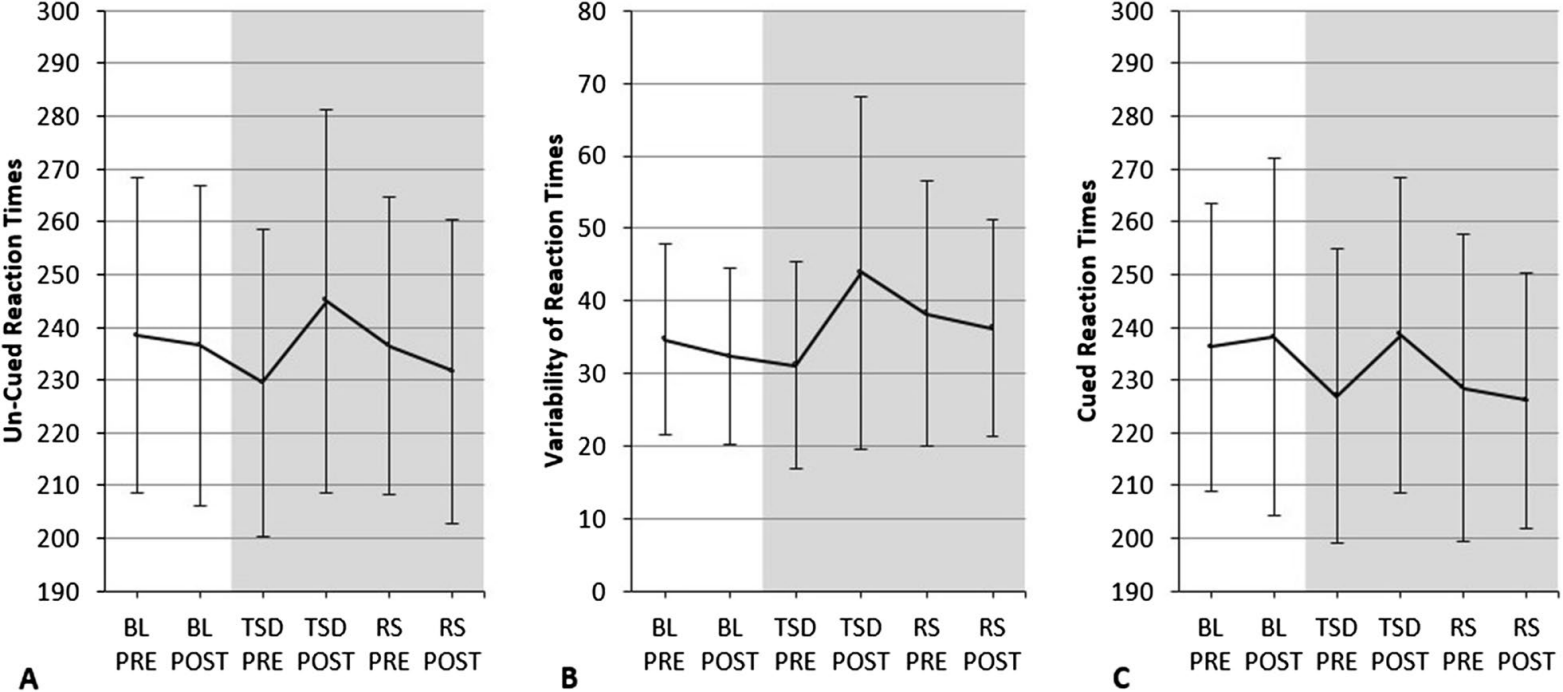
Alertness-scores.** a** Tonic alertness (reaction times (ms) in a test without warning signal). **b** Variability of reaction times in the tonic alertness test (ms). **c** Phasic alertness (reaction times (ms) in a test with warning signal). Results are presented as *M* and *SD*

Detailed alertness-scores can be found in Fig. 4. The ANOVA for tonic alertness yielded a significant interaction “condition * time of day” (*F*_2,28_ = 8.683, *p* = 0.001, *η*^2^ = 0.383). Subjects had significantly slower reaction times after sleep deprivation as compared to values assessed prior to sleep deprivation (TSD PRE vs RS PRE: *t*_29_ = − 2.724, *p* = 0.011, *d* = 0.242), but not as compared to BL-values (BL PRE vs RS PRE: *t*_29_ = 0.528, *p* = 0.601, *d* = − 0.069). Subjects’ reaction times were significantly faster after RS as compared to post-deprivation values (TSD POST vs RS POST: *t*_29_ = 3.343, *p* = 0.002, *d* = − 0.405), what indicates a restoration of reaction times after one night of recovery sleep.

Additionally, a significant interaction “condition * time of day” was found for variability of reaction times of tonic alertness (*F*_2,28_ = 9.544, *p* = 0.001, *η*^2^ = 0.405). Subjects showed enhanced attentional variability following sleep deprivation as compared to pre-deprivation values (TSD PRE vs RS PRE: *t*_29_ = − 2.557, *p* = 0.016, *d* = 0.434), but not as compared to BL-values (BL PRE vs RS PRE: *t*_29_ = − 1.107, *p* = 0.277, *d* = 0.230). Hence, subjects seemed to lose the ability to react in a stable manner after sleep deprivation (values increased by 35.88% as compared to BL-values). Values were lower after one night of recovery sleep as compared to post-deprivation values (TSD POST vs RS POST: *t*_29_ = 2.009, *p* = 0.054, *d* = − 0.383), with values decreasing to 11.97% above BL-values after a night of recovery sleep. Thus, results indicate an almost complete restoration of the stability of reaction times.

Lastly, the ANOVA for phasic alertness yielded a significant effect of factor “condition” (*F*_2,28_ = 5.822, *p* = 0.008, *η*^2^ = 0.294) as well as a significant interaction “condition * time of day” (*F*_2,28_ = 10.278, *p* < 0.001, *η*^2^ = 0.423). Cued reaction times were not significantly slower after sleep deprivation as compared to pre-deprivation values (TSD PRE vs RS PRE: *t*_29_ = − 0.699, *p* = 0.490, *d* = 0.054). However, cued reaction times were significantly faster after a night of recovery sleep as compared to post-deprivation values (TSD POST vs RS POST: *t*_29_ = 4.420, *p* < 0.001, *d* = − 0.456).

### Compliance of participants and technical soundness

To elucidate the viability of our protocol, we will give information about the compliance of participants as well as about the technical soundness.

#### Compliance of participants

The ambulant PSG-measurement to assess at-home sleep was well accepted among participants. The majority of participants was able to sleep without noticing the device; only a few reported having been occasionally aware of the device attached to the upper body or of the cables (as reported in the subjects’ morning-protocols). Instructions were well followed; only one subject had to be excluded due to oversleeping in their recovery night. The TSD-procedure was also well accepted. Some subjects preferred reading, writing or talking over playing parlor games during activity-times with a low activity-level. Further, many participants took a short walk before the morning laboratory session (TSD POST) (see Table 1). None of the subjects fell asleep during the night of monitored TSD. Lastly, the actigraphic measurement was well accepted. None of the subjects reported difficulties detaching or re-attaching the actigraph for body care.

Altogether solely one participant had to be excluded due to a non-compliance with the instructions; otherwise, participants followed the instructions well.

#### Technical soundness

Regarding the SOMNOwatch™, there were no technical failures during the at-home PSG-measurement. One subject had to be excluded due to a technical failure of the SOMNOwatch™ during actigraphy. There were no other technical failures, indicating that the device proved robust and that subjects handled it carefully and in line with the given instructions. This further underlines the viability of the device in the context of at-home recovery sleep assessments.

## Discussion

The present study aimed at validating an experimental protocol that—while meeting high methodological standards—allows an investigation of recovery sleep (RS) spent at home in a familiar sleeping environment. A second objective was to demonstrate the protocol’s applicability outside of advanced sleep laboratories. In the following paragraphs, we will summarize the evidence that the recovery sleep in our study was successfully implemented; further, we will discuss a comparison of at-home sleep (as assessed in our study) and laboratory sleep as well as considerations about technical aspects and subjects’ compliance to show the validity and viability of our protocol. Lastly, strengths and weaknesses of the presented protocol will be considered.

### Effects of experimental manipulations on sleep

Considering polysomnographically assessed sleep parameters in our healthy subjects, recovery sleep (as compared to habitual sleep) was characterized by a significantly longer total sleep time, better sleep efficiency, a faster sleep onset, a substantial SWS enhancement as well as shorter and fewer awakenings during the night. These findings—considering both general PSG parameters as well as sleep-stage specific parameters—are typical for a first night of recovery sleep after one night of acute total sleep deprivation [10–12]. In more detail, a pronounced slow wave sleep rebound was found during RS (shown as increases in both absolute and relative duration), which corresponds with previous research [10–12]. The SWS increase during RS may indicate an increased homeostatic sleep pressure counter-regulating the preceding sleep deprivation [31–35]. Generally, a compensatory increase in slow wave activity is found after a period of sustained wakefulness and a decrease after sleep; these processes are assumed to be correlates of a recovery process [36]. Slow wave sleep is hypothesized to be especially critical for a restoration [35, 37, 38] and it can be assumed that sleep deprivation might affect the restorative homeostasis mainly due to SWS deprivation [35, 36].

Lastly, considering subjective sleep quality, subjects’ self-reported overall sleep quality was significantly better during RS as compared to BL, mainly stemming from a significantly higher self-reported restfulness of sleep after the participants’ recovery night. Up to date, there are no studies available assessing self-reported subjective sleep quality after a night of recovery sleep following a night of total sleep deprivation; thus, future research should add to the findings of the present study.

In summary, the protocol for studying at-home recovery sleep used in the present study allowed for demonstrating a significant increase in objective sleep quality and sleep-stage related changes that are typical for a first night of RS after preceding acute total sleep deprivation; thus, recovery sleep was successfully implemented.

### Comparison of at-home sleep and laboratory sleep

We expected parameters of at-home sleep to not substantially differ from sleep parameters obtained in sleep laboratories. Indeed, the comparison of our sleep parameters of at-home sleep and those found in the literature in laboratory studies yielded a vast similarity.

Considering the amount of time subjects were allowed to sleep during their recovery night, our subjects were allowed to sleep longer during their recovery night at-home than it usually had been allowed in laboratory studies (typically 9 h). This resulted in a longer total sleep time during at-home recovery sleep as compared to laboratory studies, which is a notable benefit of our study design, since the allowed RS duration should not be limited to make recovery as complete as possible [2].

Regarding other general PSG parameters (sleep efficiency, sleep latency, the time subjects spent awake during the night) as well as the durations of non-REM 1 and SWS during habitual and recovery sleep, there were no marked differences between at-home and laboratory sleep. In our study, the duration of non-REM 2 was slightly longer during the subjects’ recovery night at home as compared to laboratory studies; this is likely due to the prolonged total sleep time [39] during at-home recovery sleep, which is usually accompanied by a higher amount of non-REM 2.

When comparing at-home sleep and laboratory sleep, a crucial methodological difference must be considered. In sleep laboratories, participants are usually granted an adaptation night, during which they can accustom to the unfamiliar sleeping environment and the PSG-measurement. In our study, no adaptation night was implemented. Since subjects were allowed to sleep at home in a familiar sleeping environment, they only had to adapt to the ambulant PSG-measurement. Our subjects had a relatively short sleep onset latency during their baseline-night at home as well as a good sleep efficiency, indicating that subjects slept well despite wearing the PSG-device for the first time.

In summary, despite methodological differences between at-home sleep and laboratory sleep, there were no substantial differences between at-home and laboratory sleep parameters. The most prominent finding of the comparison of at-home and laboratory sleep was a similar slow wave sleep rebound during recovery sleep.

### Effects of experimental manipulations on fatigue and alertness

Effects of at-home recovery sleep on fatigue and alertness were assessed to further validate our protocol. We hypothesized that regular recovery sleep should re-set subjective fatigue and normalize attentional deficits occurring after sleep deprivation. In the following paragraphs, results will be discussed in this regard.

Fatigue is a normal response to exertion or stress [40, 41] and can result from physiological consequences of prolonged wakefulness and inadequate sleep [42–44]. Fatigue is generally understood as a subjective experience of decrements or impairments in both physiological and psychological functioning [40, 45]. As hypothesized, fatigue was elevated after sleep deprivation, what is in line with previous studies [13, 43], and one night of recovery sleep led to a significant reduction of fatigue, indicating that a single recovery night at home with a total sleep time of (at least) 9 h is sufficient to decrease emerging fatigue and lead to a complete recovery. Thus, recovery sleep at-home re-set changes in fatigue after sleep restriction as expected.

Further, measures of alertness were objectively assessed using the Test Battery of Attentional Performance (TAP). Considering tonic alertness (test with only target but no warning stimulus), a full recovery of reaction times was found after a night of recovery sleep at home, what is in line with a previous study [10]. Considering attentional variability, subjects seemed to lose the ability to react in a stable manner after sleep deprivation; a night of recovery sleep at home then led to an almost complete recovery of attentional variability. Phasic alertness was not significantly influenced by the conducted experimental manipulations. Taken together, our results highlight that one night of at-home recovery sleep led to an almost complete return of attention to pre-deprivation levels, thus producing effects as expected and further highlighting the validity of our protocol.

### Technical soundness and subjects’ compliance

Of 33 assessed participants, only one had to be excluded due to technical failure (actigraphy). Otherwise, no severe technical problems occurred; hence, the device used for polysomnography and actigraphy proved robust and sound. Considering subjects’ compliance, the adherence to instructions was good. Subjects tolerated the implemented experimental manipulations well. Only one subject had to be excluded due to oversleeping in their recovery night. The likelihood of this failure can be reduced by providing a pre-programmed digital watch for the participants. No subject ended their study participation ahead of schedule. In light of these considerations, it can be noted that our protocol proved viable and valid to assess at-home recovery sleep.

### Strengths and weaknesses

A noteworthy strength of the present study is that subjects of a wide age range and a well-balanced male–female-ratio were assessed for a validation of the protocol. Further, the implementation of portable polysomnography is to highlight since polysomnography is seen as a gold-standard in assessing sleep and its portable version allows a thorough measurement and control of regular sleep and recovery sleep in an at-home setting. The night of total sleep deprivation was well controlled, as accomplished by a standardized procedure and a close monitoring of subjects, which guaranteed continuous wakefulness during the night. The same procedure is frequently used in laboratory sleep deprivation. Also, wrist actigraphy (as a valid tool for assessing wake and sleep episodes [46]) allowed checking whether participants stayed awake during the day following TSD.

When assessing the “recovery function” of sleep and thus checking, whether a “recovery” has taken place, it is necessary and important to compare pre-sleep and post-sleep values [34]. In our study, this was achieved by comparing data from evening and morning laboratory sessions. However, this procedure implies a few confounding factors. Pre-sleep values are influenced by preceding wake duration and wake activities; pre- and post-sleep values are both influenced by circadian rhythm [34]. Nevertheless, the implementation of pre- and post-sleep testing sessions is necessary to assess sleep-related changes in outcome variables of interest; thus, when interpreting results, the given confounding of RS-effects with time of day, circadian rhythm and amount of sustained wakefulness should be carefully taken into consideration. An empirical approach to assess pre-sleep and post-sleep values during varying times of the day can be achieved by investigating shift-workers since the times shift-workers go to bed and get up systematically vary across daytime and nighttime.

## Conclusion

In conclusion, the presented experimental protocol—as a first of its kind—proved feasible to validly implement and assess recovery sleep in a naturalistic at-home setting. The successful implementation is underlined by typical changes in sleep characteristics during RS (e.g., prolonged total sleep time, better sleep efficiency, slow wave sleep rebound) as well as a similarity of the parameters assessed in our study of at-home sleep when compared to sleep parameters of laboratory studies. Thus, the presented protocol with its easy implementation is a viable methodological tool for smaller and less-well equipped laboratories (missing the facilities of specialized sleep centers) interested in recovery sleep research. Lastly, the enhanced ecological validity originating from at-home sleep assessments as well as the fact that the present study (despite not being conducted in a specialized setting) was conducted in a well-controlled manner and yielded results comparable to those obtained in sleep laboratories remain to be highlighted.

## Data Availability

The datasets analyzed during the current study are available from the corresponding author on reasonable request.

## Abbreviations

BL: Baseline
BL POST: Laboratory session in the morning after the subjects’ baseline night at home (at 08:00)
BL PRE: Laboratory session in the evening prior to the subjects’ baseline night at home (at 18:00)
EEG: Electroencephalography
EMG: Electromyography
EOG: Electrooculography
non-REM: Non Rapid Eye Movement Sleep
POMS: Profile of Mood States
PSG: Polysomnography
PSQI: Pittsburgh Sleep Quality Index
REM: Rapid Eye Movement Sleep
RIS: Regensburg Insomnia Scale
RS: Recovery Sleep
RS POST: Laboratory session in the morning after the subjects’ recovery night at home (at 08:00)
RS PRE: Laboratory session in the evening prior to the subjects’ recovery night at home (at 18:00)
SE: Sleep Efficiency
SL: Sleep Onset Latency
SWS: Slow Wave Sleep
TAP: Test Battery of Attentional Performance
TSD: Total sleep deprivation
TSD POST: Laboratory session in the morning after the subjects’ total sleep deprivation at university (at 08:00)
TSD PRE: Laboratory session in the evening prior to the subjects’ total sleep deprivation at university (at 18:00)
TST: Total Sleep Time
